# Hypoglycaemic therapy in frail older people with type 2 diabetes mellitus—a choice determined by metabolic phenotype

**DOI:** 10.1007/s40520-022-02142-8

**Published:** 2022-06-20

**Authors:** Alan J. Sinclair, Daniel Pennells, Ahmed H. Abdelhafiz

**Affiliations:** 1 King’s College, London, UK; 2 Foundation for Diabetes Research in Older People (fDROP), Droitwich Spa, WR9 0QH UK; 3 Department of Geriatric Medicine, Rotherham General Hospital, Moorgate Road, Rotherham, S60 2UD UK

**Keywords:** Older people, Type 2 diabetes mellitus, Body composition, Hypoglycaemic therapy, Frailty, Phenotype, Management

## Abstract

Frailty is a newly emerging complication of diabetes in older people and increasingly recognised in national and international clinical guidelines. However, frailty remains less clearly defined and frail older people with diabetes are rarely characterised. The general recommendation of clinical guidelines is to aim for a relaxed glycaemic control, mainly to avoid hypoglycaemia, in this often-vulnerable group of patients. With increasing age and development of frailty, body composition changes are characterised by an increase in visceral adipose tissue and a decrease in body muscle mass. Depending on the overall body weight, differential loss of muscle fibre types and body adipose/muscle tissue ratio, the presence of any associated frailty can be seen as a spectrum of metabolic phenotypes that vary in insulin resistance of which we have defined two specific phenotypes. The sarcopenic obese (SO) frail phenotype with increased visceral fat and increased insulin resistance on one side of spectrum and the anorexic malnourished (AM) frail phenotype with significant muscle loss and reduced insulin resistance on the other. In view of these varying metabolic phenotypes, the choice of hypoglycaemic therapy, glycaemic targets and overall goals of therapy are likely to be different. In the SO phenotype, weight-limiting hypoglycaemic agents, especially the new agents of GLP-1RA and SGLT-2 inhibitors, should be considered early on in therapy due to their benefits on weight reduction and ability to achieve tight glycaemic control where the focus will be on the reduction of cardiovascular risk. In the AM phenotype, weight-neutral agents or insulin therapy should be considered early on due to their benefits of limiting further weight loss and the possible anabolic effects of insulin. Here, the goals of therapy will be a combination of relaxed glycaemic control and avoidance of hypoglycaemia; and the focus will be on maintenance of a good quality of life. Future research is still required to develop novel hypoglycaemic agents with a positive effect on body composition in frailty and improvements in clinical outcomes.

## Introduction

Worldwide, the prevalence of diabetes is increasing particularity in those above the age of 65 years and peaks (22%) at the age of 75–79 years [1]. In addition to the known diabetes-related vascular complications, diabetes appears to accelerate the emergence of frailty [2] Frailty is a dynamic state that increases vulnerability to adverse health outcomes including mortality [3]. As a result, the importance of frailty has been recognised in a number of important international clinical guidelines of diabetes management for older people [4, 5]. Clinical guidelines categorically divide older people as either robust, where tight glycaemic control is recommended, or frail where relaxed targets are preferred due to the side effects associated with hypoglycaemic therapy or where improved clinical outcomes may be considered to be less of a priority in this group of patients. For example, the recommendations for the use of the new anti-diabetes therapy of glucagon like peptide-1 receptor agonists (GLP-1RA) and sodium glucose transporter-2 (SGLT-2) inhibitors are to be only carefully considered in frail individuals due to the risk of weight loss, dehydration and hypotension [4, 5]. Also, insulin is considered as a last treatment resort, after diet and oral hypoglycaemic medications, due to the fear of hypoglycaemia in these vulnerable patients. However, up to now, clinical guidelines have been generally non-specific about frailty and are not explicit about the characterisation of these frail patients. It should be appreciated that frailty is not a single homogeneous concept and the current diagnostic tools or measures are multiple, not standardised and do not consider the metabolic side of frailty [6]. It is likely that frailty has a spectrum of different metabolic phenotypes, which may have a significant impact on the choice of the most suitable hypoglycaemic agent as well as the optimum glycaemic target [7]. The aim of this manuscript was to review the commonly used frailty measures, the characteristics of frail older people with diabetes according to their metabolic phenotype, and explore the most appropriate and suitable hypoglycaemic agents to employ to achieve optimum glycaemic targets in this group of patients.

## Methods

We undertook a detailed literature search with full assessment of relevant articles by searching the following databases: Google Scholar, Medline and Embase. We used the following Medical Subject Heading (MeSH) terms: older people, old age, elderly, diabetes mellitus, frailty, management, treatment, insulin, hypoglycaemic therapy and glucose-lowering therapy individually and in combinations. Articles were reviewed for relevance by abstract independently by the three authors. A manual search of citations in retrieved articles was performed in addition to an in-depth electronic literature search. Hand searching of relevant articles was limited by covid-19 measures and restricted access to medical libraries. We limited our selection to studies published in English language. Any disagreement between authors was resolved by consensus.

### Frailty

Frailty is defined as a state of increased vulnerability to physical or psychological stressors because of decreased physiological reserve in multiple organ systems that cause limited capacity to maintain homeostasis [8]. Frailty is neither an inevitable part of growing old nor synonymous with ageing; however, it is highly prevalent among older people [9, 10]. The prevalence of frailty increases with increasing age reaching up to 7% in people > 65 years and up to 40% in those > 80 years [11]. Frailty has significant clinical consequences that affect both older people and health care systems. For example, frail older people are at increased risk of falls, fractures and dementia that lead to disability, poor quality of life and early mortality [12–17]. These consequences are associated with an increased use of health care resources such as emergency department visits, hospitalisation and eventually institutionalisation [18]. Therefore, health care costs for frail older people are severalfold higher than non-frail individuals [19]. With the increasing age of the population, frailty will continue to have a major impact on health care systems. Several studies have demonstrated that diabetes is associated with an increased risk of frailty, which is likely to be due in part to diabetes-related complications and diabetes-associated comorbidities [20–23]. For example, hypertension and other diabetes-related complications have been shown to increase the risk and burden of frailty in older people with diabetes [22, 23]. Frailty can be screened for by applying Fried criteria which combines a total of five variables, three are self-reported (weight loss, exhaustion and reduced physical activity) and two measurements (weakness assessed by grip strength and slowness measured by gait speed) [24]. The Fried frailty phenotype independently predicted incidence of falls, worsening mobility, activities of daily living (ADL) disability, hospitalisation and mortality after 3 years of follow-up of 5,317 participants ≥ 65 years old included in the Cardiovascular health Study [24]. The Survey of Health, Ageing and Retirement in Europe (SHARE) frailty tool is similar to the Fried criteria and is validated for use in primary care [25]. For European ≥ 50 years of age, SHARE frailty tool discriminated well between frail, pre-frail and non-frail and predicted mortality among pre-frail and frail in both men and women [25]. The FRAIL scale is a tool that does not require measurements. It asks five questions, which cover Fatigue, Resistance (climbing stairs), Ambulation, number of Illnesses and Loss of weight [26]. The FRAIL scale predicts mortality and the incidence of ADL and instrumental ADL (IADL) disabilities among community-dwelling older people [26, 27]. The Clinical frailty scale (CFS) is a 9-point scale that describes patient’ functional characteristics and predicts mortality. It uses pictures that aid in stratifying patients into different levels of frailty based on their function [28]. The CFS has been widely used in clinical practice and was found to be predictive of mortality 87% of the time, associated with comorbidity 73%, complications 100%, length of hospital stay 75%, falls 71%, cognition 94% and function 91% [29]. The Frailty Trait Scale (FTS) is a short tool based on measurement of three dimensions of nutrition, physical activity and nervous system that can predict risk of hospitalisation and mortality [30]. Compared with Fried frailty phenotype tool, the FTS showed a better prediction for hospitalisation in persons ≤ 80 and for mortality in those > 80 years old [30]. The Edmonton frail scale (EFS) is another tool that does not require specialist knowledge or training in geriatric medicine [31]. EFS is associated with several geriatric conditions such independence, drugs assumption, mood, mental, functional and nutritional status [32]. The Gérontopôle frailty-screening tool (GFST) is designed to screen older people as an initial questionnaire to increase awareness of frailty [33]. The GFST showed a positive predictive value of 75.9% and a negative predictive value of 64.7% at the identification of non-disabled frail elders. These findings demonstrate an overall moderate agreement between the GFST and the Fried frailty phenotype tool [34]. The electronic frailty index (EFI) and the 35-Item Rockwood frailty index use data collected as part of comprehensive geriatric assessment and can be applied to a large number of population where their clinical data are recorded in primary care practice and a score generated via specific software [35, 36]. The EFI identified older people with mild, moderate and severe frailty and had a robust predictive validity for outcomes of mortality, hospitalisation and nursing home admission [35]. The Rockwood frailty index, when used in acuity ill patients, showed that across different levels of frailty, higher illness acuity increased mortality risk. When acuity was low, the risk was significant only when the degree of frailty was high, whereas when acuity was high, lower levels of frailty were associated with greater mortality risk [36]. The PRISMA Questionnaire is a 7-item questionnaire to identify frailty and is suitable for postal completion [37]. A cut-off score of three and above positive answers to a total seven questions revealed a sensitivity of 78.3% and a specificity of 74.7%, which might identify 35.5% of the aged people as frail [38]. The PRISMA includes a coordination-type integrated service delivery system for frail older people that showed a decreased incidence of functional decline, a decreased burden for caregivers and a smaller proportion of older people wishing to be institutionalised [37]. The main frailty assessment tools are summarised in Table 1.

**Table 1:** Frailty assessment tools

Tool	Criteria	Advantage
Fried’s phenotype. [24]	5-point scale: weight loss, exhaustion, weakness assessed by grip strength, reduced physical activity and slowness measured by gait speed	Identifies robust (score 0), pre-frail (score 1–2) and frail (score > 3) individuals but requires two practical measurements
SHARE Frailty Instrument. [25]	Five dimensions: loss of appetite, walking difficulty, exhaustion, weakness measured by grip strength and low physical activity	Proposed for the primary health care setting and accessible via web calculators
FRAIL scale. [26]	5-point scale: fatigue, resistance, ambulation, illness and loss of weight	Can be self-assessed and does not require measurements by healthcare professionals
Clinical frailty scale. [27]	9-point scale that describes patient’ functional characteristics and categorise them from very fit to severely frail	Uses clinical descriptors and pictographs to stratify older people according to level of function to predict mortality or institutionalisation
Frailty Trait Scale. [28]	Evaluates three dimensions of nutrition, physical activity and nervous system	Can predict hospitalisation and mortality
Edmonton Frail Scale. [29]	Nine domains: cognition, physical function, general health, independence, social support, pharmacological condition, nutrition, mental condition and continence	Can be completed by people without special training in geriatric medicine
Gérontopôle Frailty Screening Tool. [30]	Six questions assessing the individual’s social, physical, functional and cognitive situation	An initial screening tool in primary care to increases awareness of underlying frailty
Electronic Frailty Index. [31]	Uses the cumulative deficit model to identify and score frailty based on routine interactions of patients with their general practitioner	Can be used to screen for the whole practice population who are > 65 years old
35-Items Rockwood frailty index. [32]	35 items, based on data from chronic diseases, disabilities in activities of daily living, cognition, nutrition, visual and hearing impairment	Includes comprehensive data as a part of comprehensive geriatric assessment
PRISMA Questionnaire. [33]	7-item questionnaire to identify frailty, a score of > 3 identifies frailty	Is suitable for postal completion

### Frailty and diabetes

Diabetes is associated with an accelerated ageing process that promotes frailty, which is due in part to accelerated loss of skeletal muscles [39]. Other factors that increase the risk of frailty are the diabetes-associated complications, especially hypertension, renal impairment and dementia. In the analysis of the Mexican Health and Nutrition Survey of 7164 older people, mean (SD) age 70.6 (8.1) years, diabetes was independently associated with frailty (coefficient 0.28, *p* < 0.001) with an incremental association when hypertension (0.63, *p* < 0.001) or any diabetic complication was also present (0.55, *p* < 0.001) [22]. In a Japanese cross-sectional study of 9,606 participants ≥ 65 years of age, participants in the lowest quartile of renal function [estimated glomerular filtration rate (eGFR) < 30.0 mL/min/1.73 m^2^] showed an independent higher risk of frailty [odds ratio (OR) 1.83, 95% confidence interval (CI) 1.01 to 3.45] compared with those in the highest quartile (eGFR ≥ 60.0 mL/min/1.73 m^2^). Individuals with a history of hypertension or diabetes mellitus showed a significantly increased risk of frailty and the risk increased further when both hypertension and diabetes co-exist (OR 3.67, 95% CI 1.13–14.05) [40]. Persistent hyperglycaemia itself may be a factor that promotes frailty. In the Beijing longitudinal study of ageing II (BLSA-II) which included 10,039 subjects, mean (SD) age 70.5 (7.8) years at baseline, of whom 6,293 subjects were followed up for 12 months, the prevalence and incidence of frailty were higher in people with compared to those without diabetes (19.3% v 11.9% and 12.3% v 7.0%, respectively) and people with pre-diabetes had a similar prevalence (11.43%) but slightly higher incidence of frailty (8.7%) than people without diabetes. This suggests that the risks of frailty proportionally increase by increasing blood glucose level and pre-diabetes may play an intermediary role in the acceleration of frailty [20]. The positive correlation between frailty and hyperglycaemia (HbA1c > 6.5%) has also been shown in older women (aged 70–79 years) participating in the Women’s Health and Ageing Studies I and II. [41, 42] Several other studies have confirmed the increased risk of frailty associated with diabetes especially when diabetes-related complications are present [43–46]. Frailty is detrimental in diabetes prognosis as it increases diabetes-related complications, hospitalisation, accelerates functional decline and is associated with mortality [47]. Studies that have showed an increased risk of frailty with diabetes are summarised in Table 2. However, studies described are not easy to interpret, as they did not use a unified tool or threshold for definition or assessment of frailty. In addition, most of the studies described an association rather than a causation between diabetes and frailty, which will need further large-scale prospective studies.

**Table 2:** Recent studies exploring risk of frailty in older people with diabetes

Study	Patients	Aim to	Main findings
Castrejón-Pérez et al. cross sectional, Mexico, 2017. [22]	7164 Mexican subjects, mean (SD) age 70.6 (8.1) Y	Explore association of DM, hypertension and frailty	Independent association with frailty of:
			A. DM, hypertension or both (coefficients 0.28, 0.4 and 0.63, respectively, *p* < 0.001)
			B. Any diabetic complications, duration of DM or diabetes related physician visits (0.55, 0.01 and 0.01 respectively, *p* < 0.01)
Chhetri et al. prospective, China, 2017. [20]	10,039 subjects, mean age 70.5Y at base line, 6,293 subjects F/U 12 M	Investigate prevalence and incidence of frailty in subjects with compared to those without DM	A. Subjects with had higher prevalence (19.3% v 11.9%) and incidence (12.3% v 7.0%) of frailty compared to those without DM
			B. Prevalence risk 1.4 (95% CI 1.2 to 1.6), incidence risk 1.6 (1.3 to 1.9) in subjects with compared to those without DM
García-Esquinas et al. prospective, Spain, 2015. [43]	346 subjects with and 1,404 subjects without DM, age ≥ 60 Y, F/U 3.5 Y	Assess the incidence of frailty and possible mechanisms	A. DM increased risk of frailty (OR 2.18, 95% CI 1.42 to 3.37)
			B. Unhealthy behaviours, obesity, poor glucose control and altered serum lipid profile increased risk of frailty
			C. Diabetes nutritional therapy reduced risk of frailty
Howrey et al. prospective, US, 2018. [21]	301 subjects with and 1026 subjects without DM, age ≥ 60 Y, F/U 18 Y	Examine association of DM with odds of frailty in Mexican Americans	A. DM increased risk of frailty (OR 1.47, 95% CI 1.14 to 1.90)
			B. Other factors such as low level of education, MI, arthritis and hip fracture increased risk of frailty
Aguilar-Navarro et al. prospective, Mexico, 2015. [44]	Total 5644 participants, mean (SD) age 68.7 (6.9) Y, 11 Y F/U	Describe characteristics and prognosis of subjects classified as frail	Diabetes was significantly more common in frail than in non-frail subjects (23.7% v 9.9%, *p* < 0.001)
Castrejón-Pérez et al. cross sectional, Mexico, 2018. [23]	Total 5379 subjects, mean (SD) age 70.3 (7.8) Y	Describe associations of frailty with diabetes and related conditions in older people	A. Diabetes was associated with frailty (OR 2.32, 95% CI 1.93 to 2.73, *p* < 0.001)
			B. Most frail groups were: 1. Hospitalised in previous year (2.32, 1.69 to 3.18, *p* < 0.001)
			2. On insulin and oral therapy (5.6, 1.58 to 19.8, *p* = 0.008)
			3. Peripheral neuropathy (2.02, 1.42 to 2.86, *p* < 0.001)
Zaslavsky et al. prospective, US, 2016. [45]	Total 1848 subjects aged ≥ 65 Y, F/U 4.8 Y	Explore incidence of frailty	Incidence of frailty 37% in subjects with diabetes, 30.4% in those without diabetes (HR 1.52, 95% CI 1.19 to 1.94)
Thein et al. prospective, Singapore, 2018. [46]	Total 2696 patients aged ≥ 55 Y, 11 Y F/U	Investigate prevalence of physical frailty in subjects with compared to those without DM	Diabetes increased the risk of
			A. Physical frailty (OR 2.24, 95% CI 1.16 to 4.34)
			B. Combined physical frailty and cognitive impairment (2.01, 1.12 to 3.60)

*Y* Years, *F/U* Follow up, M Months, *DM* Diabetes mellitus, *CI* Confidence interval, *OR* Odds ratio, *MI* Myocardial infarction, *SD* Standard deviation, *HR* Hazard ratio

Frailty is associated with an increased risk of hypoglycaemia; however, the current screening tools of frailty are not able to quantify this risk. The screening tools do not consider the metabolic phenotypes of frailty or the trajectory of glycaemia. In addition, the guideline recommendations do not precisely describe the frail older people with diabetes. Frailty remains a complex and multifaceted condition. Therefore, consideration of metabolic phenotypes of frailty may help guide the choice of hypoglycaemic therapy and glycaemic targets in this heterogeneous group of patients.

### Frailty metabolic phenotypes

Skeletal muscle consists of several muscle fibres that have different metabolic properties, which may play a role in the glucose metabolism. The most clinically relevant fibres are type I or slow twitch fibres and type II or fast twitch fibres. Compared with type I, type II fibres have lower fat oxidative properties that lead to lipid storage in muscle tissue, which increase insulin resistance and glucose intolerance [48]. Therefore, type II fibres is associated with insulin resistance while type I fibres with insulin sensitivity and the predominance of one fibre or another, among other factors such as muscle mass, may influence the overall insulin sensitivity of the individual. With increasing age, there is increased atrophy of type II muscle fibres that accounts for the majority of body muscle loss [49, 50]. This may lead to a reduction in insulin resistance. Compensatory age-related increases in visceral fat and reduction in the number and function of the *β*-cells of the pancreas may lead to a general increase in insulin resistance and glucose intolerance in older age [51]. However, frailty is also associated with an accelerated muscle loss than age alone with a prominent reduction of type II than type I fibres, which may lead to an overall reduction of insulin resistance in frail older people [52–54]. The loss of muscle fibres or sarcopenia is the main characteristics of frailty and, therefore, sarcopenia and frailty can be seen as two sides of the same coin [24, 55]. Another characteristic of frailty is weight loss, although it is not an absolute necessity for frailty diagnosis and obesity can be associated with frailty [56]. Therefore, depending on overall body weight, differential loss of muscle fibres and body adipose/muscle tissue ratio, frailty can be associated with a spectrum of metabolic changes with wide variations in insulin resistance: we believe that these can be labelled as at least two distinct metabolic ‘phenotypes’. The anorexic malnourished (AM) frail phenotype with significant muscle loss and reduced insulin resistance on one side of the spectrum and the sarcopenic obese (SO) frail phenotype with increased visceral fat and insulin resistance on the other.

#### The anorexic malnourished (AM) phenotype

The coexistence of multiple comorbidities may also lead to protein energy malnutrition and muscle wasting which leads to spontaneous resolution of hyperglycaemia and reduction of HbA1c to normal ranges in older people with diabetes [57]. Normalisation of hyperglycaemia has also been shown in patients with this frailty phenotype and deintensification or even complete withdrawal of hypoglycaemic therapy was achieved without deterioration of glycaemic control [58, 59]. The main characteristics of these patients were significant weight loss and increased prevalence of comorbidities [59]. Markers of malnutrition such as low serum albumin, low cholesterol levels and weight loss have been demonstrated in participants of studies that reported an association between low HbA1c and mortality suggesting that their poor general health and frail status increased their vulnerability to adverse outcomes [47]. Therefore, age-related body composition changes such as accumulation of visceral fat, which increases insulin resistance, may be altered when anorexic-malnourished type of frailty develops. In this phenotype of frailty, a metabolic shift induced by weight loss occurs that leads to normalisation of hyperglycaemia and a change in the natural history of type 2 diabetes from a progressive to a regressive course.

#### The sarcopenic obese (SO) phenotype

Sarcopenic-obesity is an age-related muscle mass loss associated with increased visceral fat [60]. In older people, sarcopenia is closely linked to frailty and is associated with institutionalisation and mortality [61]. Sarcopenic obesity is also associated with unfavourable metabolic profile and increased risk of adverse outcomes than either obesity or sarcopenia alone [62]. In the cross-sectional analysis of 14,528 adults from the NHANES III, sarcopenic obese individuals showed the highest risk of insulin resistance and dysglycaemia [63]. Similarly, in the Korean National Health Examination and Nutrition Survey (KNHANES), which included 2943 subjects ≥ 60 years old, sarcopenic obesity was associated with insulin resistance, metabolic syndrome and dyslipidaemia [64]. One study showed that insulin resistance increased in frail older people only when abdominal obesity is present, while insulin resistance is the same in non-obese frail compared to healthy older persons [65]. Other studies have also linked sarcopenic obesity to increased risk of dyslipidaemia, diabetes mellitus and hypertension [66, 67]. Therefore, in this phenotype of frailty, the progressive course of diabetes is perpetuated. Table 3 summarises the criteria of the two main phenotypes.

**Table 3:** Frailty metabolic phenotypes

Anorexic malnourished (AM)	Sarcopenic obese (S0)
Poor appetite, reduced energy intake and weight loss	Good appetite, increased energy intake and weight gain
Reduced skeletal muscle mass and visceral fat	Reduced skeletal muscle mass and increased visceral fat
Reduced insulin resistance	Increased insulin resistance
Tendency to hypoglycaemia	Tendency to hyperglycaemia
Diabetes course is regressive	Diabetes course is progressive
Progressive deintensification of hypoglycaemic therapy	Progressive intensification of hypoglycaemic agents
Weight limiting hypoglycaemic agents are not suitable	Weight limiting hypoglycaemic agents are suitable

The above-mentioned two phenotypes are likely to be on the opposite two ends of a possible frailty metabolic spectrum that may include a graded other phenotypes. For example, some frail patients will have normal weight and normal appetite and lie in the middle of the spectrum. Another example is, while the SO phenotype is consistently insulin resistant due to obesity, the AM phenotype may be heterogeneous depending on whether the sarcopenia (which increases insulin resistance) or the weight loss (which decreases insulin resistance) is dominant. Also, patients’ clinical condition, and, therefore, their phenotype, is likely to be dynamic and change overtime. Therefore, glycaemic targets and hypoglycaemic therapy should also be dynamic and follow patient’s phenotype changes.

### Hypoglycaemic agents

In frail older people with diabetes, hypoglycaemic agents should be selected based on their risk of hypoglycaemia in addition to their cardiovascular benefits and effects on body weight. The current hypoglycaemic medications can be divided into non-hypoglycaemia inducing and hypoglycaemia inducing agents (Table 4).

**Table 4:** Special aspects of hypoglycaemic therapy in frail older people with diabetes

Agent	Benefits	Cautions	Effect on frailty
Non-hypoglycaemia inducing agents			
Metformin	Low risk of hypoglycaemia, CV protection	Lactic acidosis in patients with sepsis, organ dysfunction or dehydration. Some GI side effects. May cause vitamin B12 deficiency	May reduce the risk of frailty
DPP-4 inhibitors	Low risk of hypoglycaemia, well tolerated	GI side effects, some agents require dose adjustment in CKD, other agents may increase hospitalisation due to HF. cautions in patients with history of pancreatitis	May have a positive effect on muscle blood supply and reduction of sarcopenia
Acarbose	Low risk of hypoglycaemia may have some CV benefits	Less tolerated, GI side effects, weak hypoglycaemic effect	No data for effect on frailty
SGLT-2	Low risk of hypoglycaemia, CV and renal protection	Risk of UTI, hypotension, dehydration and candidiasis. May be associated risk of fractures and DKA	Little data, it may improve muscle quality but this not confirmed
GLP-1RA	Low risk of hypoglycaemia, CV and renal protection	GI side effects, injectable, cautions in patients with history of pancreatitis. May be associated with thyroid C-cell tumours	Little and inconsistent data
Glitazones	Low risk of hypoglycaemia, have some CV protection, suitable in CKD	Increased fluid retention, exacerbation of HF, possible increased risk of fracture and bladder cancer	May have a positive effect on muscle mass and sarcopenia
Hypoglycaemia-inducing agents			
Insulin secretagogues	Suitable in patients with CKD	High risk of hypoglycaemia	May be associated with increased risk of muscle atrophy
Insulin	Most potent hypoglycaemic agent, suitable in patients with organ dysfunction	High risk of hypoglycaemia, injectable and burden of blood glucose monitoring	Anabolic effect may improve muscle mass but further research still required

*CV* Cardiovascular, *GI* Gastrointestinal, *DPP-4* Dipeptidyl peptidase, *CKD* Chronic kidney disease, HF Heart failure, *SGLT-2* Sodium glucose transporter, *GLP-1RA* glucagon like peptide-1 receptor agonists, *UTI* Urinary tract infection, *DKA* Diabetic ketoacidosis

#### Non-hypoglycaemia inducing agents

Metformin offers both low risk of hypoglycaemia and cardiovascular (CV) benefits. A meta-analysis has shown that metformin was associated with lower long-term (≥ 2 years) CV mortality compared with sulfonylureas [hazard ratio (HR) 0.6–0.7 and 0.6–0.9 from randomised controlled trials (RCT) and observational studies respectively] [68]. Metformin reduction of CV events has also been demonstrated in patients with chronic comorbidities [69]. Metformin is generally weight neutral but it may promote some weight loss probably through its anorectic effect [70]. Metformin was associated with lower risk of frailty (OR 0.66, 95% CI 0.61–0.71, *p* < 0.0001) compared with sulfonylureas [71]. Metformin was also associated with a delayed onset of age related comorbidities including dementia, depression and frailty in a large prospective study of 41,204 men with type 2 diabetes, mean age (SD) 74.6 (5.8) years, followed up for over 9 years [72]. The DPP-4 inhibitors have low risk of hypoglycaemia, well tolerated and are weight neutral although they lack the CV benefits of metformin [73]. Clinical trials showed that DPP-4 inhibitors did not increase the risk of the composite CV end points and the risk of hospitalisation due to congestive heart failure (CHF) was inconsistent. The HR was significant for saxagliptin (1.27, 95% CI 1.07–1.51), marginally increased, but was not significant, for alogliptin (1.07, 95% CI 0.79–1.46) and not significant for sitagliptin [1.00 (0.83–1.20)] [74–76]. A meta-analysis of DPP-4 inhibitors trials has shown overall CV safety but non-significant increases in heart failure, especially with saxagliptin [77]. Because of the controversy of the heart failure hospitalisation risk observed with some DDP-4 inhibitors apart from linagliptin and sitagliptin, caution should be considered when using such agents especially there is, so far, no clear definition of the patients at risk for this detrimental effect and its mechanisms are still unknown. DPP-4 inhibitors may have a beneficial effect on the reduction of lean muscle loss although there are no clinical trials yet available [78]. The alpha glucosidase inhibitor, acarabose, delays carbohydrate absorption in the gastrointestinal tract slowing the spike in postprandial blood glucose. Although it can cause diarrhoea, it may have some CV benefits, a low risk of hypoglycaemia and it promotes weight loss [79]. The new agents of SGLT-2 inhibitors and GLP-1RAs have novel mechanisms of action that promote weight loss with low risk of hypoglycaemia. The SGLT-2 inhibitors reduce proximal tubular reabsorption of glucose leading to increased glucosuria, attenuation of hyperglycaemia and reduction of body weight. The GLP-1 RAs stimulate post-prandial insulin secretion, which is glucose-sensitive, therefore the overall risk of hypoglycaemia is low. Clinical trials have demonstrated that the risk of hypoglycaemia of these agents is comparable to that of placebo [80–88]. In addition, these agents have demonstrated a consistent and significant cardio-renal protective effect [89]. They may also have a positive effect on liver functions in patients with diabetes and non-alcoholic fatty liver disease (NAFLD) and non-alcoholic steatohepatitis (NASH) [90]. The efficacy and safety of these newer agents appear to include older people (≥ 65 years of age) who represented almost 50% of the participants in these trials [89]. The effects of these agents on frailty are still not clear. In a Japanese study which included a total of 194 patients with diabetes, mean age 65.7 years, the prevalence of patients with high risk of falling (defined as a weaker hand grip and a shorter duration of one leg standing) was significantly higher in those treated with GLP-1 RAs compared to other hypoglycaemic medications (45.7% v 27.5%). This may suggest that GLP-1 RAs may increase the risk of frailty [91]. The SGLT-2 inhibitors luseogliflozin and canagliflozin have shown a reduction of skeletal muscle mass, which may increase the risk of sarcopenia and frailty [92–94]. However, another study showed that dapagliflozin did not reduce muscle mass [95]. In addition, it has been reported that SGLT-2 inhibitors improve muscle quality rather than muscle mass which may have an overall positive effect [96]. Glitazones are associated with low risk of hypoglycaemia but increased weight gain, likely related to a combination of fluid retention and redistribution of adipose tissue [97]. The glitazone, pioglitazone reduces the risk of major CV events but increases the risk of heart failure and peripheral oedema [98]. Glitazones, through their insulin sensitising properties, may have beneficial effects on reducing the loss of lean muscle mass [99]. In the follow-up phase of the study of osteoporotic fractures which included a total of 2864 community dwelling women, mean (SD) age 78.5 (3.6) years, insulin sensitiser treatment (metformin/glitazones) had preserved walking speed compared with other treatment (*p* < 0.05) [100] However, glitazones are associated with bone loss in older women with type 2 diabetes and possibly associated with an increased risk of fracture independent of age and gender [101, 102].

#### Hypoglycaemia inducing agents

There is little literature on the CV safety of insulin secretagogues such as sulfonylureas or glinides. They are associated with increased risk of hypoglycaemia, weight gain and their CV effects are not very clear [79]. There was no difference in CV risk when sulfonylurea was added to metformin but significantly lower risks of acute myocardial infarction were found for the glinides plus metformin [adjusted hazard ratio (HR) 0.39, 95% confidence interval (CI) 0.20 to 0.75] [79]. The available data for gliclazide and repaglinide on CV outcomes are limited but other agents such as glimepiride, glibenclamide, glipizide and tolbutamide may be associated with an increased risk of all-cause mortality and increased risk of muscle atrophy [103, 104]. The evidence from clinical trials and observational studies suggests an increase in all-cause and/or CV mortality associated with insulin secretagogues, whereas the increase in the incidence of major CV events in patients treated with secretagogues, which is usually observed in epidemiological studies, is not similarly evident in randomized clinical trials. Among secretagogues, those with a lower affinity for myocardial ATP-dependent potassium channels, such as gliclazide, could be associated with a lower mortality than glibenclamide; however, these differences, reported in some observational studies, have never been confirmed by randomised trials [105]. The advantage of sulphonylurea and glinides is that they can be used in patients with impaired renal function although careful monitoring for hypoglycaemia will be required. Insulin remains the most effective hypoglycaemic therapy but is associated with increased risk of hypoglycaemia that may increase the risk of falls and significant weight gain [73]. A recent meta-analysis has concluded that treatment with insulin increased the risk of fractures among patients with type 2 diabetes compared with oral anti-diabetic drugs however, the association was influenced by various other factors such as gender, fracture site, study design and geographical region [106]. Insulin appears to have a neutral effect on CV outcomes [107]. Insulin has been shown to stimulate muscle protein synthesis in younger but not in older persons and has not been shown to reduce muscle atrophy in diabetes [108].

### Hypoglycaemic therapy

**Fig. 1:**
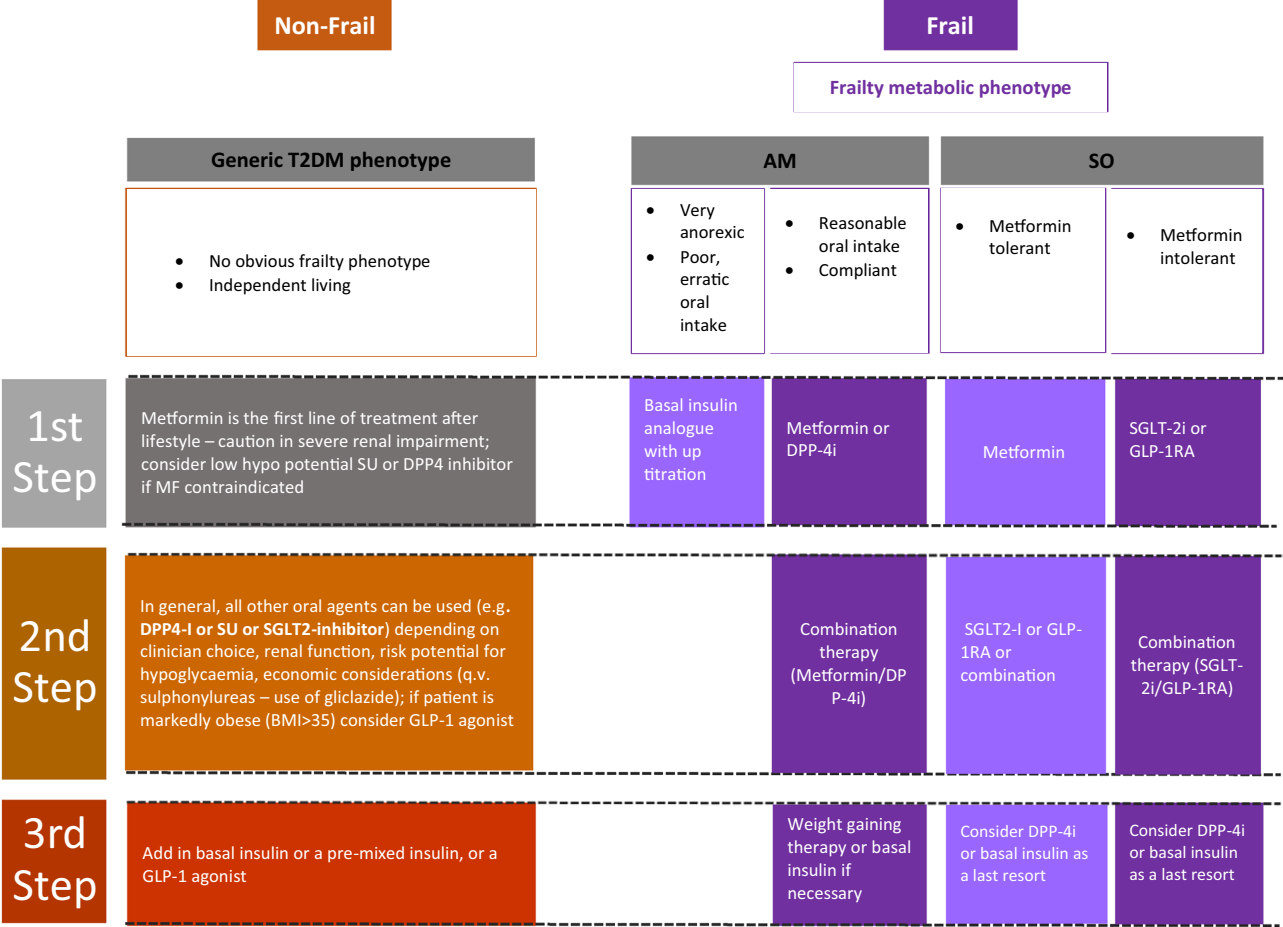
Step-by-step approach to glucose lowering in type 2 diabetes in older people: a general metabolic phenotype compared with two metabolic phenotypes of frailty. *AM* Anorexic malnourished, *SO* Sarcopenic obese, *DPP-4i* Dipeptidyl peptidase-4 inhibitors, *SGLT-2i* Sodium glucose transporter-2 inhibitors, *GLP-1RA* Glucagon-like peptide-1 receptor agonists. *T2DM* type 2 diabetes mellitus

So far, there are no hypoglycaemic medications specifically designed for older people with comorbid diabetes and frailty. The general recommendation is to avoid the risk of hypoglycaemia as well as the cautious use of the new therapies, GLP-1RA and SGLT-2 inhibitors, to reduce the risk of hypotension, dehydration, weight loss and falls in this vulnerable population [4, 5]. However, frailty is not a homogeneous condition and frailty phenotypes should be taken into consideration when deciding which hypoglycaemic agent is best for this group of patients. Therefore, we suggest a pragmatic approach that considers hypoglycaemic agents that promote weight loss to be the first choice in the SO phenotype and weight promoting or neutral agents, including insulin, to be considered in the AM phenotype. In addition to weigh-based hypoglycaemic choice, other important aspects such as cardiovascular protection especially in high risk SO phenotype and quality of life/avoidance of hypoglycaemia especially in the AM phenotype should be considered. In addition, the use of low (sub-maximal) doses of oral hypoglycaemic agents in combined format can be useful to reduce side effects while preserving a sufficient efficacy. In Fig. 1, for comparison purposes, we align a standard step-by-step approach to glucose lowering in older people (the general phenotype) with the approach we are suggesting could be used for managing the two distinct metabolic phenotypes we have categorised [109].

#### The sarcopenic obese phenotype

Metformin remains the first line therapy in this phenotype due to its cardiovascular benefits and safety profile. The new therapy of GLP-1RAs and SGLT-2 inhibitors should be used early in this phenotype as a second line after metformin or first line if metformin is not tolerated or contraindicated. The efficacy of these new therapies extends to include older age groups as demonstrated in clinical trials [82–88]. For example, the post-hoc analyses of the EMPA-REG OUTCOME study (44.6% of participants ≥ 65 years) and of the DECLARE study (46% of participants ≥ 65 years) have found that the risks of cardiovascular mortality, heart failure and renal outcomes were reduced across all age groups [110, 111]. In the age-stratified meta-analysis of the SGLT-2 inhibitors clinical trials, the cardiovascular benefits were consistent across all age groups [112]. Similarly, the post-hoc analysis of the LEADER study (75% of participants ≥ 60 years old) has found that patients aged ≥ 75 (only 9%) had a 34% risk reduction in the frequency of major adverse cardiovascular events (MACE) in the liraglutide intervention arm compared with placebo arm (HR 0.66, 95% CI 0.49 to 0.89, *p* = 0.006). The SUSTAIN-6 study of semaglutide which included 43% of participants > 65 years old has shown similar results [113, 114]. Although this data is reassuring, the lack of frailty assessment on inclusion of the participants makes it uncertain whether these new therapies are safe in this vulnerable group. However, given that > 50% of the participants are > 65 years old and have multiple comorbidities is assuring. Another advantage of these new therapies is their extra-glycaemia effect, which will improve the metabolic profile of these sarcopenic obese individuals by reducing their body weight, visceral fat and increasing their insulin sensitivity. Recent studies have shown that these new therapies to have beneficial effects on NAFLD and its progression to NASH in patients with type 2 diabetes and a significant decrease in hepatic fat content [115, 116]. In addition, GLP-1RA may have the potential to improve neurodegenerative conditions such as Alzheimer’s and Parkinson disease as well as bone density in older people with diabetes [117, 118]. With the use of these new therapies in sarcopenic obese frail individuals with multiple comorbidities, it may be possible to de-intensify the prescription of other medications such as diuretics and antihypertensives which may lead to a reduction of polypharmacy and its negative consequences on therapy burden, adverse drug events and medication non-compliance. If glycaemia is not yet controlled, DPP-4 inhibitors can be used as add on therapy if required while other weight gaining agents or insulin should be the last resort.

#### The anorexic malnourished phenotype

This frail phenotype is likely to be in the oldest age group with multiple comorbidities, polypharmacy and less tolerance to drug therapy likely due to associated organ dysfunction. In the milder form of this phenotype such as people who are compliant with oral therapy and nutrition, metformin or DPP-4 inhibitors can be first line therapy, mainly due to their lower risk of hypoglycaemia. GLP-1RAs and SGLT-2 inhibitors are not suitable in this phenotype due to the undesirable associated weight loss induced with these agents. Insulin secretagogues, although they have the advantage of desirable weight gain in this phenotype, they should be avoided due to their high risk of hypoglycaemia. Insulin should be considered early on in this phenotype, especially in those who are less compliant with oral therapy and have significant weight loss. Insulin therapy could produce a sustained improvement in the older people well-being [119]. Weight gain associated with insulin will be an advantage in this group of patients but other insulin-associated side effects such as the inconvenience of frequent injections, blood glucose monitoring and the increased risk of hypoglycaemia should be considered. It has been shown that early introduction of insulin to existing oral hypoglycaemic medications to be more effective (HbA1c reduction of 1.5% with insulin vs 0.6% with increased oral doses) and have less hypoglycaemic events (23 vs 79, *p* = 0.03) than further increasing the oral doses indicating that adding insulin early on may be a safer option than increasing oral hypoglycaemic agents. [120] Reduced frequency of insulin injections and simplicity of titration are desirable features for patients' compliance, quality of life and the reduction of the risk of hypoglycaemia. Long-acting analogues should always be preferred to NPH human insulin for the lower risk of hypoglycaemia. The most convenient and simple regimen is the long acting basal insulin at bedtime because of its effectiveness, simplicity and only once-daily dosing. For example, the use of long acting insulins (glargine or detemir) has been shown to reduce emergency department visits or hospitalisation due to hypoglycaemia compared with NPH insulin in older people with type 2 diabetes [121]. The ultra-long acting basal insulin degludec demonstrates a flat and stable glucose lowering effect with once-daily administration [122]. The smoother pharmacokinetic and pharmacodynamic profiles of degludec insulin may reduce the frequency and magnitude of blood glucose troughs, thereby reducing the frequency and severity of hypoglycaemic episodes. It has been shown that insulin degludec achieves glycaemic control that is comparable to, or better than, that of insulin glargine with significantly lower rates of overall or nocturnal hypoglycaemia [123]. Once daily basal insulin should be used first but it will ameliorate the nocturnal hepatic glucose production with no much effect on post-prandial blood glucose levels. In patients with markedly reduced insulin secretion, an insufficient insulin response to meals can produce a selective post-prandial glucose increase, and attempt at countering this defect with a basal insulin alone may have a limited efficacy and it may determine a high risk of inter-prandial hypoglycaemia, particularly in those with irregular meals. Although many frail people will do well with this non-physiological regimen, there will be a need to consider adding prandial or mealtime short acting insulin cover when persistent postprandial glucose excursions occur. However, to reduce the risk of hypoglycaemia especially in patients with erratic eating pattern, short acting insulin analogue should always be preferred to regular human insulin for flexibility in the timing of administration, efficacy on postprandial glucose and lower risk of late postprandial hypoglycaemia and preferably administered after a meal is consumed [124].

### Glycaemic control

There are no large clinical trials to investigate the effect of glycaemic control on frailty as a main outcome. It appears that dysglycaemia (both hyperglycaemia and hypoglycaemia) increases the risk of frailty although the mechanism of this is poorly understood [3]. Frailty due to persistent hyperglycaemia could be attributed to skeletal muscle mitochondrial dysfunction, microvascular damage, hyperglycaemia-related complications or other mechanisms such as increased inflammation and increased oxidative stress [125–127]. On the other hand, hypoglycaemia may increase the risk of frailty by inducing repeated minor subclinical cerebral injuries or recurrent falls and fractures that may, over time, lead to functional impairment [124]. Therefore, the ideal short-term glycaemic control is to avoid the wide excursions in blood glucose levels to reduce the time patients spent in dysglycaemia. Zaslavsky et al. have found a U-shaped relationship between blood glucose levels and the risk of incident frailty with blood glucose levels < 160 mg/dL and > 180 mg/dL to be associated with increased risk of frailty (*p* = 0.001). [45] The ideal long-term glycaemic control or HbA1c is less clear. A previous study has shown that HbA1c ≥ 8.0 to be associated with low walking speed while HbA1c < 7% was associated with better lower extremity performance [128]. Also, in a study of 5,035 older people with a mean age of 75.0 years, those with HbA1c > 7.0% had a significantly higher prevalence of functional disability [129]. However, other studies did not demonstrate a beneficial effect of tight glycaemic control on physical function and it has been associated with an increased risk of hypoglycaemia, falls and fractures [130]. The U-shaped relationship demonstrated by Zaslavsky et al. has also found that HbA1c of 7.6% to be associated with the lowest risk of frailty with a HR (95% CI) of 1.41 (1.12 to 1.78) for HbA1c of 6.9% and 1.30 (1.08 to 1.56) for HbA1c of 8.2%. [45] Therefore, HbA1c around a target of 7.5% may be a reasonable target to reduce the risk of frailty in most older people with diabetes. However, a target range of 7.0–8.5% mmol/mol, based on severity of frailty, has been suggested [4].

### Education and glucose monitoring

Older people with diabetes may tolerate lower blood glucose with less specific symptoms of hypoglycaemia due to diminished autonomic response [131, 132]. Therefore, educational programmes are important for patients and their carers. For example, in a study that delivered a diabetes educational programme to care home staff, staff knowledge improved and was retained at 12 months and led to improved quality of care for residents with diabetes up to a year after the intervention [133]. Monitoring of blood glucose is an integral part of diabetes management that is crucial to achieve adequate glycaemic control and avoid hypoglycaemia. Self-monitoring of blood glucose (SMBG) may be useful in patients on insulin therapy or those on oral hypoglycaemic agents with high hypoglycaemic risk potential or during acute illness otherwise, its value is less clear [134]. It may have value in the initial titration of hypoglycaemic therapy on diagnosis but it is likely not to be required as a long-term monitoring tool, to avoid frequent finger pricking and maintain quality of life, in most patients with type 2 diabetes and stable glycaemia [134]. Continuous glucose monitoring (CGM) is another technology that adds more information about the time spent in the target range or the severity, frequency and duration of hyper and hypoglycaemic episodes [135]. Relaxed glycaemic targets are not an assurance of a lower risk of hypoglycaemia as CGM has unmasked frequent episodes of hypoglycaemia in older people with higher HbA1c levels [136]. In type 2 diabetes mellitus, CGM is suitable in patients on insulin or oral therapy with significant glycaemic variability [137]. CGM has been shown to be associated with a reduction of diabetes-related stress and an improvement in well-being [138]. Glucose monitoring will need informed discussion between clinicians and patients with particular attention to patient goals to avoid unnecessary burden and to maintain quality of life. In addition, physical and cognitive functions of patients and their carers should be considered when planning glucose monitoring.

### Goals of therapy

**Fig. 2:**
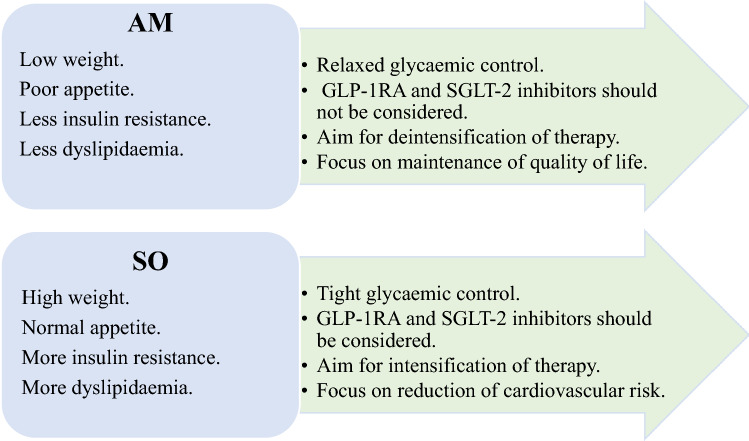
Goals of therapy in the two metabolic phenotypes of frailty in older people with diabetes. *AM* Anorexic malnourished, *SO* Sarcopenic obese, *GLP-1RA* Glucagon-like peptide-1 receptor agonists, *SGLT-2* Sodium glucose transporter-2

An important goal of therapy was to achieve the best glycaemic control possible with the minimum side effects. The goals of therapy should be tailored to each metabolic phenotype. It has been shown that lower HbA1c (< 7%) to be associated with increased mortality risk compared with moderate levels (≥ 7% < 8.5%) in patients using regimens that are associated with hypoglycaemia [139]. High levels of HbA1c were consistently associated with elevated mortality risk in those regimens that have a lower risk of hypoglycaemia. These data suggest that, in the individualisation of glycaemic targets, consideration needs to be given to the classes of glucose-lowering therapy that are being used, with less aggressive targets in those patients who are being treated with therapies associated with hypoglycaemia [139]. Goals of therapy are, therefore, depends on the metabolic phenotype of the frail older patient with diabetes (Fig. 2).

#### The sarcopenic obese phenotype

In this phenotype, diabetes follows a progressive course and obesity is commonly associated with other cardiovascular risk factors such as dyslipidaemia, hypertension and insulin resistance. Therefore, a key goal of therapy in this phenotype is to reduce the cardiovascular risk and achieve tight glycaemic control without increasing the risk of hypoglycaemia. The general recommendation by the guidelines for relaxed targets in frail older people should not be applicable to this metabolic phenotype. Lower HbA1c was associated with increased mortality risk compared with moderate control in those regimens associated with hypoglycaemia [139]. Therefore, the use of GLP-1RAs and SGLT-2 inhibitors are ideal in this phenotype due their lower risk of hypoglycaemia and their properties of reducing cardiovascular risk early on therapy independent of glycaemic control. Therefore, reasonable intensification of therapy and reduction of CV risk is a main goal in this phenotype.

#### The anorexic malnourished phenotype

In this phenotype, diabetes follows a regressive course due to anorexia and significant weight loss. As a result, this phenotype will have less insulin resistance, blood glucose level decline and may lead to spontaneous resolution of hyperglycaemia and normalisation of HbA1c [57]. This group of patients are likely to be very frail with a limited life expectancy. The general recommendation by the guidelines for relaxed targets in frail older people are applicable to this metabolic phenotype. The use of weight limiting agents especially GLP-1RA and SGLT-2 inhibitors should be avoided. As insulin is likely to be used early on in this phenotype, relaxed targets are appropriate as aggressive lowering of HbA1c with hypoglycaemic agents with high hypoglycaemia risk may be associated with increased mortality [139]. Therefore, deintensification of therapy and a focus on good quality of life are main goals of therapy in this metabolic phenotype.

## Summary and conclusion

Frailty is a new emerging complication of diabetes in older people. Clinical guidelines are based on an individual’s physical function with recommendations of tighter glycaemic control in the fit individuals and relaxed targets in those with frailty. However, frail older people are metabolically heterogeneous and further research may allow recommendations to be tailored to suit key frailty metabolic phenotypes as we have proposed. For the sarcopenic obese (SO) phenotype, weight limiting hypoglycaemic agents, especially the new agents of GLP-1RA and SGLT-2 inhibitors, should be considered early on in therapy due to their benefits on weight reduction and cardiovascular protection that is independent of glycaemic control. Goals of therapy is tight glycaemic control, without inducing hypoglycaemia and the focus will be on the reduction of their cardiovascular risk. In the anorexic malnourished (AM) phenotype, weight neutral or insulin therapy, should be considered early on in therapy due to the benefits of avoiding weight loss and possible anabolic and weight gaining effects of insulin. Goals of therapy is relaxed glycaemic control, avoidance of hypoglycaemia and the focus will be on maintenance of good quality of life.

### Future perspectives

In routine clinical practice, even in specialist centres, frailty still does not seem to be taken into consideration when deciding on diabetes management, glycaemic control and HbA1c targets [140, 141]. We have demonstrated that frailty is a complex metabolic condition with a spectrum of metabolic phenotypes with variation in insulin sensitivity that may affect the choice of hypoglycaemic therapy. This view applied to frailty is similar conceptually to a recent Scandinavian study, which identified 5 different subtypes of patients with type 2 diabetes that have different characteristics, insulin resistance, disease progression and risk of diabetes-related complications—these concepts form the basis of future ‘precision’ medicine [142]. Therefore, in future clinical trials, older participants should not be defined by age alone but frailty phenotype should also be clearly characterised. However, the relationship of frailty and diabetes is complex as frailty, although a complication associated with diabetes, it can be present before the onset of diabetes. In addition, the spectrum of metabolic profile of frailty is likely to be more complex. For example, some patients will be frail with normal weight and normal appetite in the middle of the spectrum. Another example is, while the sarcopenic obese phenotype is consistently insulin resistant due to obesity, the anorexic phenotype may be heterogeneous depending on whether the sarcopenia (which increases insulin resistance) or the weight loss (which increases insulin sensitivity) is dominant. In addition, patients with diabetes and mild obesity may have no evidence of insulin resistance or malnutrition but the prevalence and the incidence of frailty in this category is unknown. Another future perspective is the effect of hypoglycaemic agents on frailty. So far, there is very little literature on the effect of these agents on frailty or body composition. For example, although there is some evidence to suggest that SGLT-2 inhibitors may induce diabetes-associated sarcopenia, other studies did not confirm these findings [143–149]. Also, pioglitazone has been shown to potentiate the effect of resistance training on muscle strength in women but not in men and the effect of DPP-4 inhibitors and GLP-1RAs on body composition is not consistent. Therefore, the current data on this issue is limited and future trials are warranted [150–155]. The anabolic properties of insulin and its effect on body muscle needs further exploration. The new insulin analogues appear as a potentially favourable therapy in the AM frail phenotype as long as hypoglycaemia is avoided. It may have the potential to improve the muscle mass and increase the body weight in this frail phenotype. Insulin stimulates muscle protein synthesis and anabolism in younger individuals but this anabolic effect is blunted in older people, which may suggest that higher doses of insulin are required to achieve this anabolic effect [156, 157]. Previous study have shown a positive effect of insulin on skeletal muscle index and improvement of sarcopenia in the lower extremities in a relatively younger 312 participants with type 2 diabetes, mean (SD) age 64 (11) years [158]. However, in the recent population-based KORA-Age study which included 118 older people with type 2 diabetes, mean (SD) age 74.6 (6.2) years, insulin therapy was associated with preserved muscle mass, but not muscle function parameters [159]. These finding suggest that further trials are still required to fully investigate the anabolic effect of insulin in frail older people with diabetes.
